# Paraurethral Endometrioid Carcinoma Arising From Ectopic Endometriosis: A Case Report

**DOI:** 10.1002/iju5.70255

**Published:** 2026-09-27

**Authors:** Keita Ogasawara, Takashi Tsuyukubo, Mizuki Hisano, Hiromitsu Fujisawa, Shigekatsu Maekawa, Mitsugu Kanehira, Mitsumasa Osakabe, Naoki Yanagawa, Hiroaki Itamochi, Wataru Obara

**Affiliations:** ^1^ Department of Urology Iwate Prefectural Central Hospital Iwate Japan; ^2^ Department of Urology Iwate Medical University School of Medicine Iwate Japan; ^3^ Department of Diagnostic Pathology Iwate Medical University School of Medicine Iwate Japan; ^4^ Department of Clinical Oncology Iwate Medical University School of Medicine Iwate Japan

**Keywords:** carcinoma, dysuria, endometrioid, endometriosis, lymphatic metastasis

## Abstract

**Introduction:**

Malignant transformation of endometriosis is rare and most commonly involves the ovary; transformation from paraurethral endometriosis is exceedingly rare.

**Case Presentation:**

A 73‐year‐old postmenopausal woman with prior ovarian endometrioma presented with dysuria and voiding difficulty. Positron emission tomography–computed tomography revealed a paraurethral mass at the bladder neck, whereas cystoscopy and urine cytology were normal. Transvaginal biopsy demonstrated adenocarcinoma; however, immunohistochemistry did not identify the primary site. After radical cystectomy with urethrectomy and partial vaginectomy, histopathology showed endometrioid carcinoma arising from paraurethral endometriosis with lymph node metastasis. Comprehensive genomic profiling identified alterations consistent with endometrioid/Müllerian‐type carcinoma and compatible with endometriosis‐associated carcinogenesis. Despite chemotherapy and immune checkpoint inhibitors, the patient died approximately 16 months after diagnosis.

**Conclusion:**

Paraurethral endometrioid carcinoma is exceptionally rare. Accurate diagnosis relies on careful correlation of imaging with histopathology, while comprehensive genomic profiling provides corroborative molecular evidence.

## Keynote Message

A symptomatic paraurethral mass in a postmenopausal woman can represent endometrioid carcinoma arising from ectopic endometriosis. Diagnosis requires careful correlation of imaging findings with histopathology.

## 1 Introduction

Endometriosis affects 6%–10% of women of reproductive age [1]. Malignant transformation is rare and most commonly involves the ovary, presenting as endometrioid or clear cell carcinoma [2].

Extraovarian transformation is less frequent, occurring in the pelvis, bowel, abdominal wall, and urinary tract [2, 3], and may carry a higher risk in postmenopausal women [4]. Within the urinary tract, the bladder and ureter are the most common sites, whereas paraurethral involvement is exceedingly rare [3, 5].

Here, we describe a rare case of endometrioid carcinoma arising from paraurethral ectopic endometriosis in a postmenopausal woman.

## 2 Case Presentation

A 73‐year‐old postmenopausal woman who underwent right oophorectomy for ovarian endometriosis approximately 30 years earlier presented with persistent dysuria and voiding difficulty unresponsive to antibiotics for presumed cystitis. Pelvic examination revealed a palpable paraurethral mass. Pelvic magnetic resonance imaging showed a 36‐mm paraurethral mass at the bladder neck displacing the anterior vaginal wall and compressing the urethra without intraluminal involvement (Figure 1a–d). Positron emission tomography–computed tomography demonstrated intense uptake confined to the paraurethral lesion (maximum standardized uptake value, 13.5) (Figure 1e,f). No abnormality of the uterus, peritoneal lesions, ascites, or lymphadenopathy was detected on cross‐sectional imaging, and the posterior vaginal wall and rectum were intact. The uterus and the remaining left adnexa were preserved at surgery and not sampled.

**FIGURE 1:**
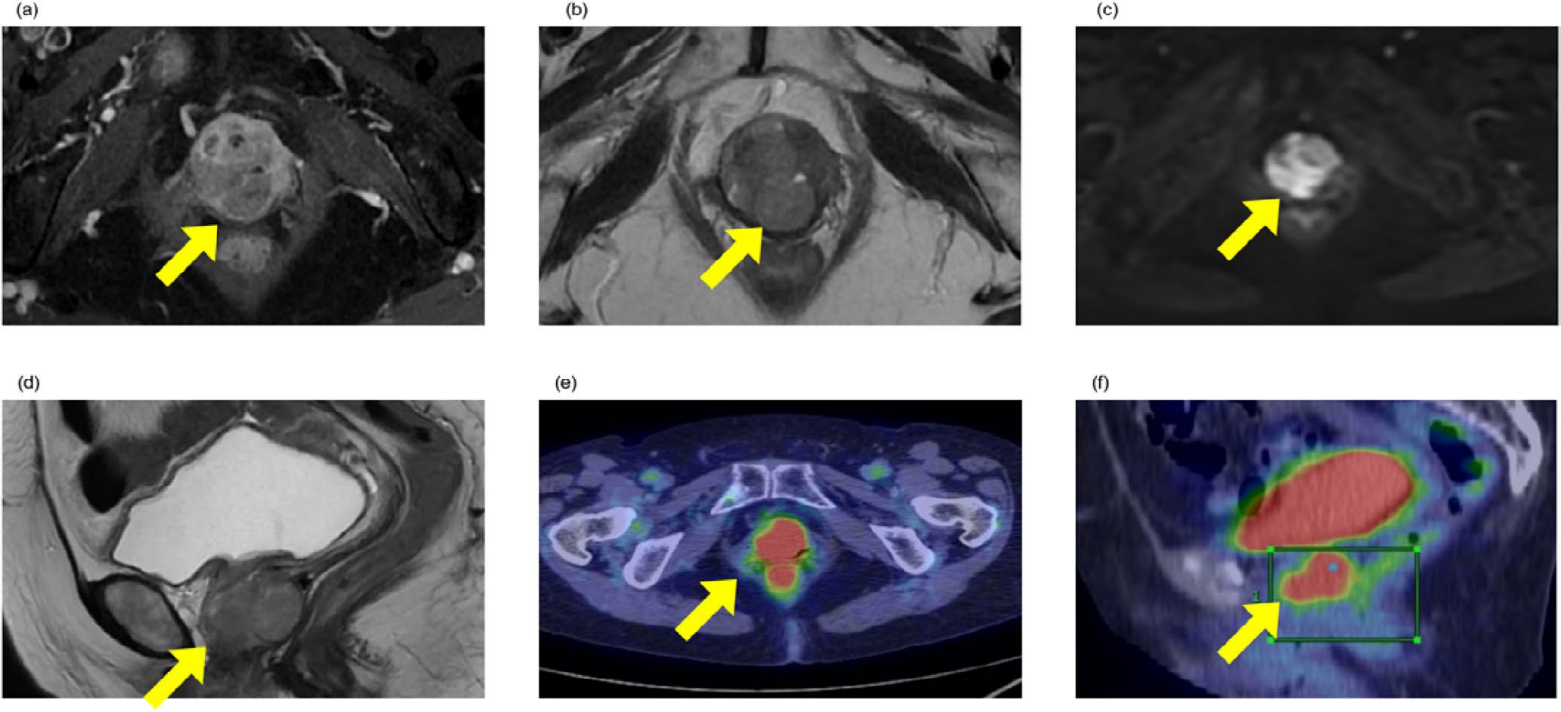
Pelvic magnetic resonance imaging (MRI) and fluorodeoxyglucose positron emission tomography–computed tomography findings of the paraurethral mass. (a) Axial T1‐weighted MRI shows a well‐circumscribed paraurethral mass compressing the urethra without intraluminal involvement (arrow). (b) Axial T2‐weighted MRI shows a 36‐mm paraurethral mass (arrow). (c) Diffusion‐weighted MRI demonstrates restricted diffusion within the lesion (arrow). (d) Sagittal T2‐weighted MRI shows a mass beneath the bladder neck, displacing the anterior vaginal wall without a definite invasion of the bladder or uterus (arrow). (e, f) Axial and sagittal fluorodeoxyglucose positron emission tomography–computed tomography fusion images demonstrate intense focal uptake in the paraurethral lesion (arrows; maximum standardized uptake value, 13.5). MRI, magnetic resonance imaging.

Cystoscopy showed no intravesical tumor or urethral diverticulum, and urine cytology was negative; however, vaginal‐wall cytology was positive for malignant cells. A transvaginal incisional biopsy demonstrated adenocarcinoma; immunohistochemistry supported this diagnosis but did not identify the primary site (Table S1). Evaluation for an occult primary tumor—including gastrointestinal endoscopy, transvaginal ultrasonography, and serum tumor markers—revealed no gastrointestinal neoplasm or endometrial thickening; endometrial biopsy was not performed, and serum CA125 and carcinoembryonic antigen levels were elevated (Table S2). No primary malignancy was identified in the uterus, ovaries, or gastrointestinal tract. Although no direct invasion of the bladder or urethral urothelium was identified, the tumor extended to the bladder neck and periurethral soft tissue, making bladder‐sparing resection unlikely to achieve negative margins. Given the patient's and her family's wish to avoid long‐term catheterization, she underwent radical cystectomy with urethrectomy, partial vaginectomy, ileal conduit urinary diversion, and pelvic lymph node dissection. Because the uterus was uninvolved, anterior exenteration was not required (Figure 2a,b).

**FIGURE 2:**
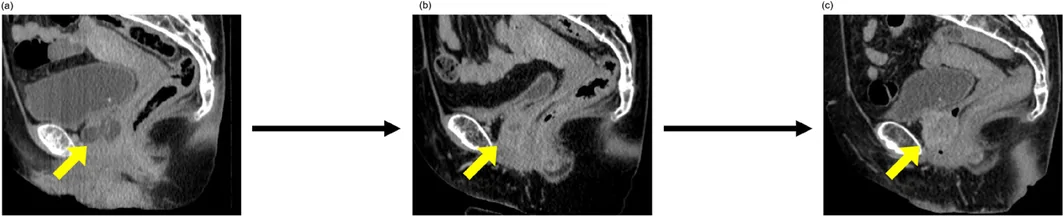
Gross and histopathological findings of paraurethral endometrioid carcinoma arising from ectopic endometriosis. (a) En bloc surgical specimen following radical cystectomy with urethrectomy and partial vaginectomy; the paraurethral lesion is indicated (yellow box). (b) Enlarged view of the boxed area in (a); the urethral lumen is outlined in cyan, and the periurethral tumor on either side of the urethral lumen is outlined in magenta. (c) Hematoxylin and eosin (HE)‐stained section of the transition zone; boxes 1–3 correspond to panels (d, e), (f), and (g), respectively. (d) Region 1: Endometriosis with tubal‐type epithelial metaplasia (HE, ×40). (e) Serial section corresponding to region 1: CD10 immunostaining labels the endometrial‐type stroma beneath the surface epithelium (×40). (f) Region 2: Atypical hyperplasia with nuclear enlargement and prominent nucleoli (HE, ×40). (g) Region 3: Grade 1 endometrioid carcinoma with a confluent cribriform pattern (HE, ×20). These findings demonstrate a histological transition from endometriosis through atypical hyperplasia to carcinoma. Scale bars: 500 μm (c), 50 μm (d–f), and 100 μm (g).

Histopathological examination showed Grade 1 endometrioid carcinoma arising within ectopic endometrial glands and stroma in the paraurethral tissue, contiguous with benign endometriosis, with a histological transition from endometriosis through atypical hyperplasia to carcinoma (Figure 2c–g). The tumor expressed PAX8 and cytokeratin 7 with wild‐type p53, and the endometrial‐type stroma was positive for CD10 (Table S1). The initial biopsy, obtained during the occult‐primary work‐up, had suggested poorly differentiated adenocarcinoma, but central review showed uniformly Grade 1 endometrioid carcinoma, with no high‐grade, dedifferentiated, or mixed component in the entire resection specimen (Figure S1). The tumor was centered in the paraurethral submucosa, and the bladder and urethral urothelium were unremarkable. Together with the positive vaginal cytology, this distribution supported a paraurethral rather than a vaginal mucosal origin. Lymphovascular invasion was present, with a positive soft‐tissue margin and a negative urethral margin; 2 of 18 obturator lymph nodes were involved (right, 2 of 6), without extranodal extension.

Adjuvant chemotherapy was recommended because of the positive surgical margin and nodal disease, but the patient declined. Adjuvant radiotherapy was also declined. Approximately 6 months after surgery, computed tomography revealed new supraclavicular, para‐aortic, and iliac lymph node metastases, indicating systemic rather than local progression. Paclitaxel and carboplatin plus bevacizumab were administered for recurrent disease, followed by nivolumab. The disease progressed despite treatment, and the patient developed grade 3 hypertension and proteinuria (treatment doses, cycles, response, and toxicities are shown in Table S2). Comprehensive genomic profiling (FoundationOne CDx; Foundation Medicine, Cambridge, MA, USA) revealed truncating variants in PTEN, ARID1A, and BRCA1, a high tumor mutational burden (27 mutations per megabase), and microsatellite‐stable status. These findings were consistent with endometrioid/Müllerian‐type carcinoma and compatible with endometriosis‐associated carcinogenesis (Table 1). The patient died approximately 16 months after the initial diagnosis.

**TABLE 1:** Genomic profiling by FoundationOne CDx and report‐annotated U.S. Food and Drug Administration (FDA)–approved therapeutic options.

Detected somatic mutation/biomarker	FDA‐approved therapeutic option(s)
Tumor mutational burden (TMB), 27 mutations/Mb (TMB‐high)	Pembrolizumab
Microsatellite status (MS), microsatellite stable (MSS)	—
BRCA1 (Q1200)	—
AKT2 (amplification)	—
ARID1A (Q211)	—
CCNE1 (amplification)	—
NF1 (S2587)	—
PTEN (Q245)	—
STK11 (K78N)	—
AXL (amplification)	—
CDKN2A (exon 1 rearrangement)	—
KMT2D (splice‐site variant, c.4584‐2delA)	—
PBRM1 (E1226)	—

*Note:* FDA‐approved therapeutic options are as annotated in the FoundationOne CDx report. A dash (—) indicates that no FDA‐approved option was listed for that alteration.

Abbreviations: CDx, companion diagnostic; FDA, Food and Drug Administration; MS, microsatellite; MS‐stable, microsatellite stable; muts/Mb, mutations per megabase; TMB, tumor mutational burden; TMB‐high, high tumor mutational burden.

## 3 Discussion

To our knowledge, malignant transformation of paraurethral endometriosis has not been reported. Endometrioid adenocarcinoma of the urethrovaginal septum has been described, but without coexisting endometriosis [6]; the present case is distinguished by carcinoma arising within histologically contiguous paraurethral endometriosis.

Extraovarian endometriosis‐associated malignancies, including those of the urinary tract, are rare [2, 3, 7, 8]. Urethral involvement is uncommon [9, 10, 11], and paraurethral endometriosis is particularly rare, with only seven benign cases described in a recent review [5]. A paraurethral mass is generally benign—a urethral diverticulum, Skene duct cyst, Gartner duct cyst, or leiomyoma—and therefore malignancy may be overlooked. Normal cystoscopic findings and negative urine cytology do not exclude malignancy. Retrospective imaging suggested a previously cystic paraurethral lesion developed solid components before diagnosis (Figure 3), although the mechanisms and risk factors for malignant transformation at this site remain poorly understood.

**FIGURE 3:**
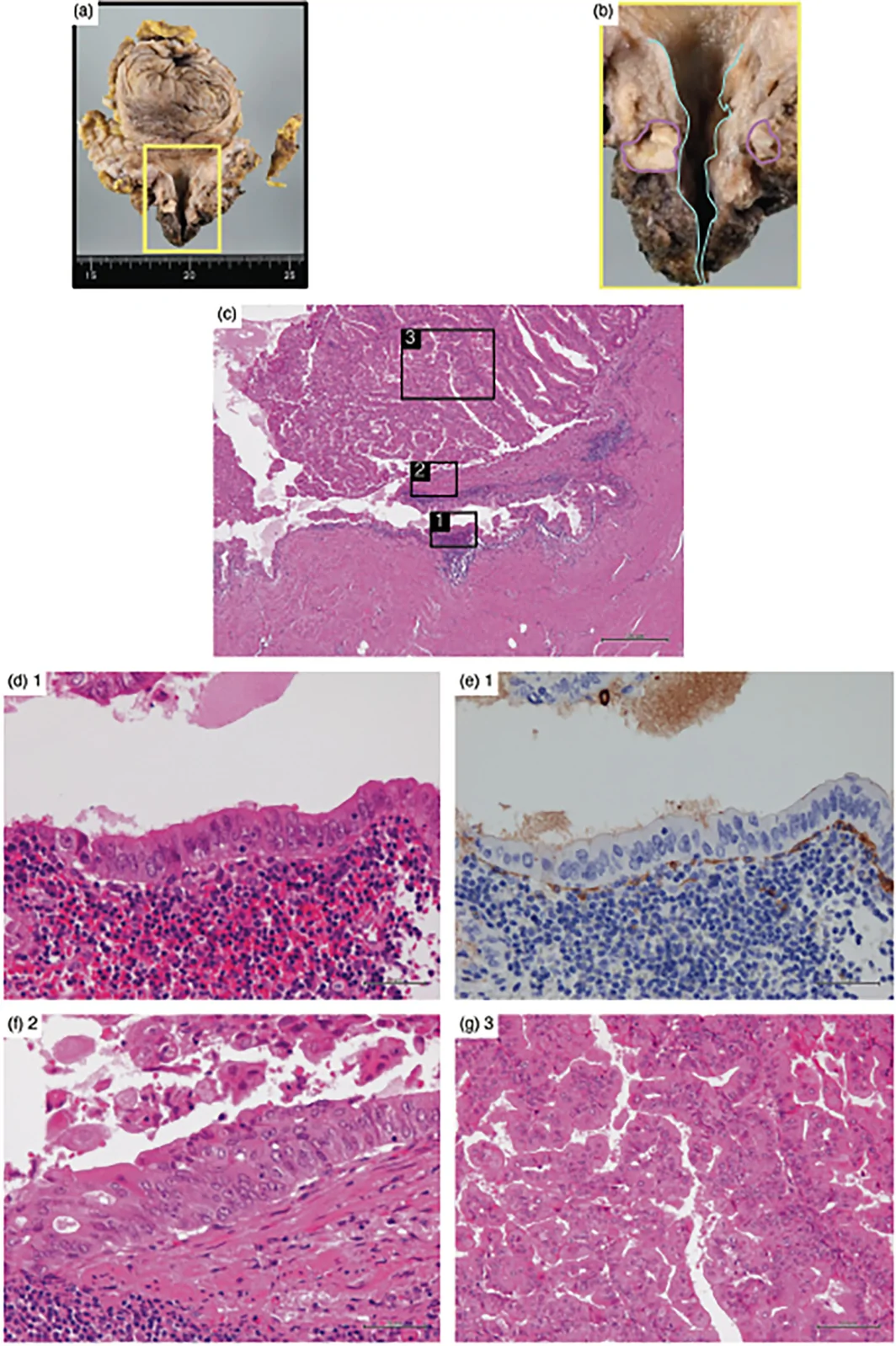
Retrospective review of serial pelvic computed tomography demonstrating stepwise change of a paraurethral lesion. Serial pelvic computed tomography images show a cystic paraurethral lesion 16 years before diagnosis (a, arrow), subsequent development of a mural solid component 11 years before diagnosis (b, arrow), and progression to a solid mass at diagnosis (c, arrow), later confirmed as endometrioid carcinoma.

Malignant transformation of endometriosis is conventionally evaluated using the Sampson criteria—endometriosis adjacent to the tumor, exclusion of a metastatic tumor, and the presence of endometrial‐type glands and stroma—and the additional Scott criterion of a histological transition from benign endometriosis to carcinoma [12, 13, 14]. The present case fulfilled the first three criteria: endometrial‐type stroma adjacent to the carcinoma (CD10‐positive), no evidence of an occult primary tumor, and contiguity with benign endometriosis (Figure 2c–g). A histological transition from endometriosis through atypical hyperplasia to carcinoma was also observed, satisfying the additional Scott criterion. Not all of these criteria are considered obligatory [14].

Comprehensive genomic profiling identified PTEN and ARID1A alterations, frequently reported in endometriosis‐associated carcinomas [2, 15]. Although the tumor mutational burden was high, its predictive value for response to immune checkpoint inhibitors varies across tumor types [16, 17, 18]. After progression on chemotherapy, nivolumab was selected instead of tumor mutational burden‐based pembrolizumab according to Japanese reimbursement criteria, supported by a phase II trial in cancer of unknown primary; however, no clinical benefit was observed [19].

This report has several limitations. A uterine or other Müllerian primary was excluded radiologically but not histologically because an endometrial biopsy was not performed and the uterus was preserved. The remaining adnexa and peritoneum were not sampled; therefore, concurrent endometriosis could be excluded only based on imaging findings. Comprehensive genomic profiling used tumor tissue alone, and the PTEN and ARID1A alterations do not localize the primary site (the PTEN variant was subclonal). Accordingly, these molecular findings should be regarded as corroborative rather than diagnostic. In addition, HE4 and TFPI‐2 levels were not measured.

In summary, endometrioid carcinoma can arise from paraurethral endometriosis and may present as a symptomatic paraurethral mass in postmenopausal women. An enlarging paraurethral lesion or one that develops new solid components warrants biopsy and cross‐sectional imaging, even when cystoscopic findings and urine cytology are unremarkable. Accurate diagnosis depends on correlating cross‐sectional imaging with histopathology, whereas comprehensive genomic profiling provides corroborative molecular evidence when standard systemic treatment options are limited. Additional cases are needed to define the optimal management of this rare entity.

## Consent

Written informed consent was obtained from the patient for the publication of this case report and accompanying images.

## Conflicts of Interest

The authors declare no conflicts of interest.

## Supporting information


**Table S1:** Immunohistochemistry (IHC) findings (biopsy and resection/central review).
**Table S2:** Clinical course, systemic therapy regimens, doses, toxicities, and serial serum tumor marker levels.
**Figure S1:** Histological grade of the paraurethral endometrioid carcinoma. (a) Low‐power view of the transvaginal biopsy (HE, ×4); boxes b and c indicate the fields shown in (b) and (c). (b) A well‐differentiated area with well‐formed endometrioid glands in the biopsy (HE, ×20). (c) A poorly differentiated area of predominantly solid growth (solid nests) in the biopsy, on the basis of which the biopsy was reported as poorly differentiated adenocarcinoma (HE, ×20). (d) A solid focus in the resection specimen; across the whole tumor such solid areas constituted ≤ 5% (HE, ×20). Architectural grading of the entire resection specimen showed uniform Grade 1 endometrioid carcinoma in which nuclear atypia was not severe, and no high‐grade, dedifferentiated, or mixed component was identified. HE, hematoxylin and eosin Histological grade and the biopsy‐to‐resection sampling difference. Endometrioid carcinoma is graded by architecture—the proportion of solid (non‐glandular) growth: Grade 1, well‐formed glands predominate with solid nests comprising ≤ 5%; Grade 2, > 5%–50%; Grade 3, > 50% (a grade may be raised one step for severe nuclear atypia). The transvaginal biopsy, obtained during the work‐up for an occult primary tumor, contained both a well‐differentiated area with well‐formed glands (b) and a poorly differentiated area of predominantly solid growth (c); on the basis of the latter, the biopsy was reported as poorly differentiated adenocarcinoma. On architectural grading of the entire resection specimen, well‐formed glands predominated at low power (c), solid nests comprised ≤ 5% (a representative solid focus is shown in d), and nuclear atypia was not severe, establishing a uniform Grade 1; no high‐grade, dedifferentiated, or mixed component was identified. The small biopsy over‐represented a focally solid area, whereas grade reflects the proportion of solid growth across the whole tumor, which was ≤ 5% (Grade 1). Figure 2 additionally documents the malignant transformation—the histological transition from endometriosis through atypical hyperplasia to carcinoma (the Scott criterion).

## Data Availability

The data that support the findings of this study are available from the corresponding author upon reasonable request.
