# Supplementary material for: Paraurethral Endometrioid Carcinoma Arising From Ectopic Endometriosis: A Case Report
Source: IJU Case Rep. 2026 Sep 27;9(6):e70255. doi: 10.1002/iju5.70255 (PMC13617304; doi:10.1002/iju5.70255)
Supplement: Supplementary file 1 [file IJU5-9-e70255-s001.zip › iju570255-sup-0001-TableS1-S2-FigureS1@5236731_5_Supplementary_Material_dedup_20260820.docx]

Supplementary Tables S1 & S2 and Figure S1

# Table S1. Immunohistochemistry (IHC) findings (biopsy and resection/central review)

**(A) Markers supporting endometrioid / Müllerian differentiation**

| **Marker** | **Biopsy** | **Resection/Central review** | **Interpretation** |
| --- | --- | --- | --- |
| PAX8 | Not performed | Positive | Müllerian differentiation |
| Cytokeratin 7 | Positive (diffuse) | Positive | Glandular/Müllerian |
| Vimentin | Not performed | Positive (focal) | Consistent with endometrioid |
| CA125 | Not performed | Positive | Müllerian differentiation |
| CD10 (stroma) | Not performed | Positive (cyst wall) | Endometrial-type stroma |
| Estrogen receptor | Not performed | Negative | Hormone-receptor-negative; atypical for low-grade endometrioid, but diagnosis supported by PAX8+/vimentin+/morphology, and adjacent endometriosis |
| Progesterone receptor | Not performed | Negative | Hormone-receptor-negative |

**(B) Markers used to exclude differential diagnoses**

| **Marker** | **Biopsy** | **Resection/Central review** | **Interpretation** |
| --- | --- | --- | --- |
| GATA3 | Negative | Negative | Excludes urothelial or breast |
| p63/p40 | p40-negative | p63-negative | Excludes urothelial or squamous differentiation |
| PSA | Not performed | Negative | Excludes Skene gland/prostatic |
| Cytokeratin 20 | Positive (very focal) | Negative | Argues against a lower gastrointestinal |
| CDX2 | Positive (weak) | Positive (focal) | Not a diffuse intestinal staining pattern |
| TTF-1 | Not performed | Negative | Excludes lung |
| Napsin A | Positive (very focal) | Not performed | Argues against clear cell |
| p53 | Not performed | Wild-type | Argues against serous |

**(C) Reviewer-requested markers not performed (covered by other markers or comprehensive genomic profiling)**

| **Marker** | **Status** | **Rationale for omission** |
| --- | --- | --- |
| Uroplakin II/III | Not performed | Urothelial origin was already excluded (GATA3, p63, and cytokeratin 20 were all negative) |
| SATB2 | Not performed | Cytokeratin 20-negative with only weak CDX2 → not intestinal |
| HNF1β | Not performed | Morphology + focal Napsin A exclude clear-cell |
| PSAP | Not performed | PSA-negative excludes Skene-gland/prostatic |
| MMR (MLH1/PMS2/MSH2/MSH6) | Not performed | Microsatellite-stable by comprehensive genomic profiling |

**(D) Mechanistic markers — immunohistochemistry not performed**

| **Marker** | **Status** | **Note** |
| --- | --- | --- |
| ARID1A (IHC) | Not performed (validated antibody unavailable) | Mechanistic marker (R1-6, optional); the ARID1A genomic alteration is reported by comprehensive genomic profiling (Table 1) |
| PTEN (IHC) | Not performed (validated antibody unavailable) | Mechanistic marker (R1-6, optional); the PTEN genomic alteration is reported by comprehensive genomic profiling (Table 1) |

*Footnote S1:* The biopsy specimen was additionally negative for SMA, desmin, MyoD1, S100, Melan A, CD34, CD31, and cytokeratin 5/6, excluding sarcomatous, melanocytic, and vascular differentiation. Staining intensity or localization was recorded where applicable (diffuse, focal, or very focal; for example, cyst wall for stromal staining); however, the proportion of positive cells (%) was not recorded separately.

# Table S2. Clinical course, systemic therapy regimens, doses, toxicities, and serial serum tumor marker levels

**(a) Timeline (approximate, relative to diagnosis [Month 0])**

| **Time** | **Event** |
| --- | --- |
| Month 0 | Diagnosis: paraurethral mass on imaging; transvaginal biopsy revealed adenocarcinoma |
| ~Month 2 | Radical cystectomy + urethrectomy + partial vaginectomy + ileal conduit + pelvic lymph node dissection (uterus preserved); positive soft-tissue margin, negative urethral margin, obturator nodal metastasis |
| ~Month 3 | Postoperative carcinoembryonic antigen nadir; adjuvant chemotherapy and radiotherapy declined |
| ~Month 8 (≈6 months after surgery) | Recurrence: supraclavicular, para-aortic, and iliac nodal metastases; comprehensive genomic profiling submitted |
| ~Months 8–11 | Paclitaxel + carboplatin + bevacizumab (panel b) |
| ~Months 13–15 | Nivolumab (panel b) |
| ~Month 16 | Death (palliative care) |

**(b) Systemic therapy**

| **Regimen** | **Dose** | **Schedule/Cycles** | **Modification** | **Best response** | **Key toxicity (CTCAE grade)** |
| --- | --- | --- | --- | --- | --- |
| Paclitaxel + carboplatin + bevacizumab | Paclitaxel 175 mg/m^2^; carboplatin AUC 6; bevacizumab 15 mg/kg | Every 3 weeks, 4 cycles | Bevacizumab withheld in cycles 3–4 for grade 3 hypertension and proteinuria; cytotoxic agents at full dose | Nodal shrinkage with a new soft-tissue lesion → progressive disease (RECIST, descriptive; no formal target-lesion measurement) | hypertension grade 3; proteinuria grade 3; thrombocytopenia grade 4 (platelet nadir 18,000/µL); anemia grade 3 (hemoglobin nadir 6.6 g/dL); course complicated by disseminated intravascular coagulation and bilateral pyelonephritis |
| Nivolumab | 240 mg (fixed dose) | Every 2 weeks, 4 cycles | — | Progressive disease; no clinical benefit | — |

**(c) Serum tumor marker levels by disease phase**

| **Marker (Unit)** | **Presentation** | **Postoperative nadir** | **Recurrence** | **During treatment → progression** |
| --- | --- | --- | --- | --- |
| Carcinoembryonic antigen (ng/mL) | 327.7 | 6.4 | 264.0 | Declined to 169.0 on chemotherapy, then increased to 778.0 on nivolumab |
| CA125 (U/mL) | 174.3 | — | 218.0 | — |
| CA19-9 (U/mL) | 19.4 | — | 162.0 | — |

*Footnote S2:* AUC, area under the concentration–time curve; CTCAE, Common Terminology Criteria for Adverse Events; RECIST, Response Evaluation Criteria in Solid Tumours. HE4 and TFPI-2 were not measured.

# Supplementary legends (for the end of the main paper)

- **Figure S1.** Histological grade of the paraurethral endometrioid carcinoma. (a) Low-power view of the transvaginal biopsy (HE, ×4); boxes b and c indicate the fields shown in (b) and (c). (b) A well-differentiated area with well-formed endometrioid glands in the biopsy (HE, ×20). (c) A poorly differentiated area of predominantly solid growth (solid nests) in the biopsy, on the basis of which the biopsy was reported as poorly differentiated adenocarcinoma (HE, ×20). (d) A solid focus in the resection specimen; across the whole tumor such solid areas constituted ≤5% (HE, ×20). Architectural grading of the entire resection specimen showed uniform Grade 1 endometrioid carcinoma in which nuclear atypia was not severe, and no high-grade, dedifferentiated, or mixed component was identified. HE, hematoxylin and eosin.
- **Histological grade and the biopsy-to-resection sampling difference.** Endometrioid carcinoma is graded by architecture—the proportion of solid (non-glandular) growth: Grade 1, well-formed glands predominate with solid nests comprising ≤5%; Grade 2, >5%–50%; Grade 3, >50% (a grade may be raised one step for severe nuclear atypia). The transvaginal biopsy, obtained during the work-up for an occult primary tumor, contained both a well-differentiated area with well-formed glands (Fig. S1b) and a poorly differentiated area of predominantly solid growth (Fig. S1c); on the basis of the latter, the biopsy was reported as poorly differentiated adenocarcinoma. On architectural grading of the entire resection specimen, well-formed glands predominated at low power (Fig. 2c), solid nests comprised ≤5% (a representative solid focus is shown in Fig. S1d), and nuclear atypia was not severe, establishing a uniform Grade 1; no high-grade, dedifferentiated, or mixed component was identified. The small biopsy over-represented a focally solid area, whereas grade reflects the proportion of solid growth across the whole tumor, which was ≤5% (Grade 1). Figure 2 additionally documents the malignant transformation—the histological transition from endometriosis through atypical hyperplasia to carcinoma (the Scott criterion).
