# Supplementary material for: Paraurethral Endometrioid Carcinoma Arising From Ectopic Endometriosis: A Case Report
Source: IJU Case Rep. 2026 Sep 27;9(6):e70255. doi: 10.1002/iju5.70255 (PMC13617304; doi:10.1002/iju5.70255)
Supplement: Supplementary file 1 [file IJU5-9-e70255-s001.zip › iju570255-sup-0002-FigureS1@5236731_Figure_S1.pdf]

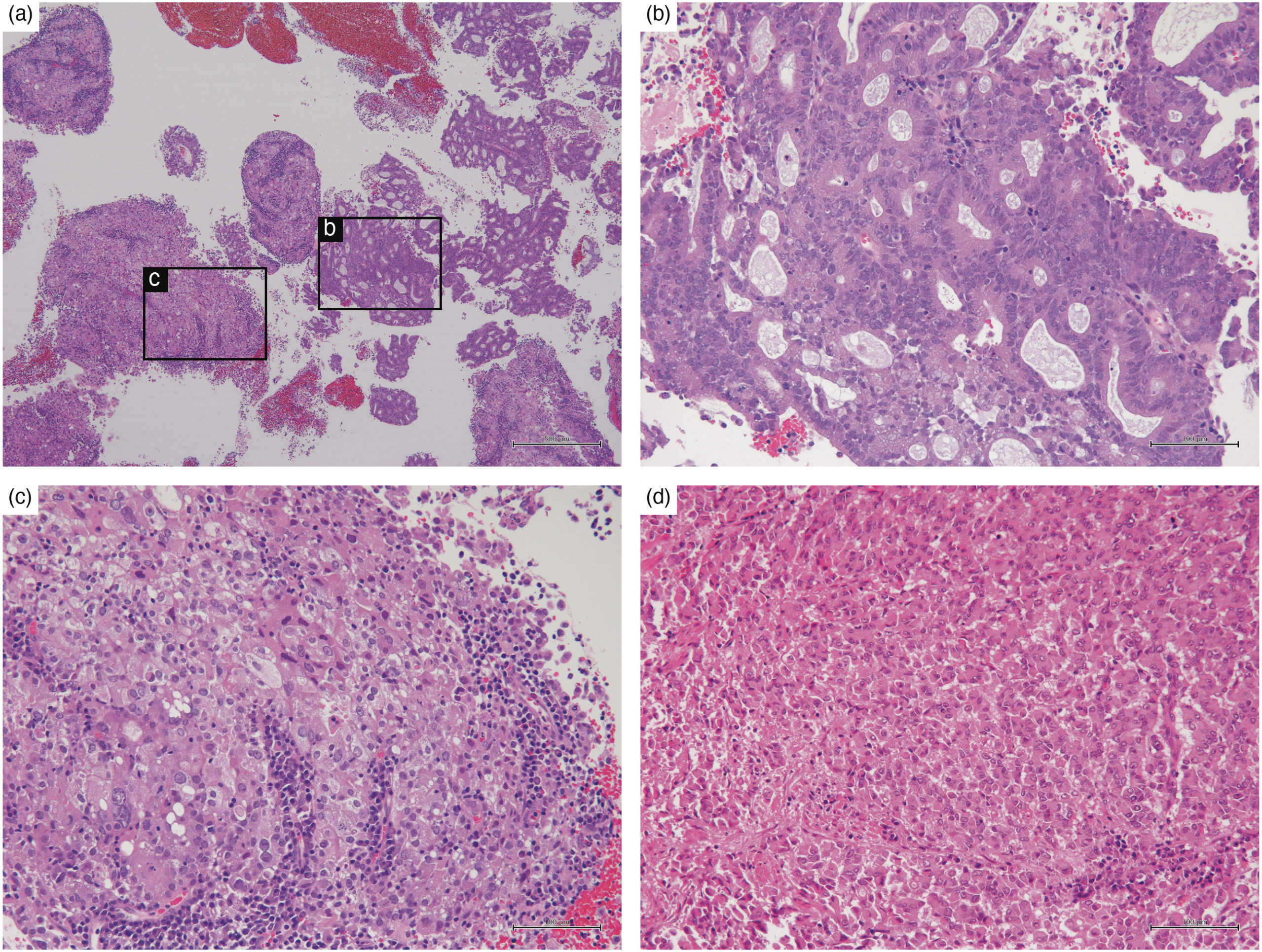

(a)
b
c
(b)
(c)
(d)
